# Association of dietary patterns with serum phosphorus in maintenance haemodialysis patients: a cross-sectional study

**DOI:** 10.1038/s41598-020-68893-4

**Published:** 2020-07-23

**Authors:** Ban-Hock Khor, Ayesha Sualeheen, Sharmela Sahathevan, Karuthan Chinna, Abdul Halim Abdul Gafor, Sunita Bavanandan, Bak-Leong Goh, Zaki Morad, Zulfitri Azuan Mat Daud, Pramod Khosla, Angela Yee-Moon Wang, Tilakavati Karupaiah, PaTCH Investigators, Boon Cheak Bee, Ghazali Ahmad, Soo Kun Lim, Mohammad Zaimi Abdul Wahab, Ravindran Visvanathan, Rosnawati Yahya

**Affiliations:** 1 Department of Medicine, Faculty of Medicine, Universiti Kebangsaan Malaysia, Cheras, 56000 Kuala Lumpur, Malaysia; 2 Dietetics Program, Faculty of Health Sciences, Universiti Kebangsaan Malaysia, 50300 Kuala Lumpur, Malaysia; 3 School of Medicine, Faculty of Health and Medical Science, Taylor’s University Lakeside Campus, Subang Jaya, 47500 Selangor, Malaysia; 4 Department of Nephrology, Hospital Kuala Lumpur, 50586 Kuala Lumpur, Malaysia; 5 Clinical Research Center, Hospital Serdang, 43000 Kajang, Selangor Malaysia; 6 National Kidney Foundation Malaysia, 46100 Petaling Jaya, Selangor Malaysia; 7 Department of Nutrition and Dietetics, Faculty of Medicine and Health Science, Universiti Putra Malaysia, Seri Kembangan, 43400 Selangor, Malaysia; 8 Department of Nutrition and Food Science, Wayne State University, Detroit, MI 48202 USA; 9 Department of Medicine, Queen Mary Hospital, The University of Hong Kong, Hong Kong, China; 10 School of BioSciences, Faculty of Health and Medical Science, Taylor’s University, Lakeside Campus, Subang Jaya, 47500 Selangor, Malaysia; 11 Department of Nephrology, Hospital Selayang, Batu Caves, 68100 Selangor, Malaysia; 12 Department of Nephrology, Universiti Malaya Medical Center, 59100 Kuala Lumpur, Malaysia

**Keywords:** Nephrology, Kidney diseases, Nutrition

## Abstract

Sources of dietary phosphate differentially contribute to hyperphosphatemia in maintenance haemodialysis (MHD) patients. This cross-sectional study in Malaysia investigated association between dietary patterns and serum phosphorus in MHD patients. Dietary patterns were derived by principal component analysis, based on 27 food groups shortlisted from 3-day dietary recalls of 435 MHD patients. Associations of serum phosphorus were examined with identified dietary patterns. Three dietary patterns emerged: *Home foods* (HF_dp_), *Sugar-sweetened beverages* (SSB_dp_), and *Eating out noodles* (EO-N_dp_). The highest tertile of patients in HF (T3-HF_dp_) pattern significantly associated with higher intakes of total protein (*p* = 0.002), animal protein (*p* = 0.001), and animal-based organic phosphate (*p* < 0.001), whilst T3-SSB_dp_ patients had significantly higher intakes of total energy (*p* < 0.001), inorganic phosphate (*p* < 0.001), and phosphate:protein ratio (*p* = 0.001). T3-EO-N_dp_ patients had significantly higher intakes of total energy (*p* = 0.033), total protein (*p* = 0.003), plant protein (*p* < 0.001), but lower phosphate:protein ratio (*p* = 0.009). T3-SSB_dp_ patients had significantly higher serum phosphorus (*p* = 0.006). The odds ratio of serum phosphorous > 2.00 mmol/l was significantly 2.35 times higher (*p* = 0.005) with the T3-SSB_dp_. The SSB_dp_ was associated with greater consumption of inorganic phosphate and higher serum phosphorus levels.

## Introduction

Hyperphosphatemia is associated with increased mortality and morbidity in maintenance haemodialysis (MHD) patients^1,2^. The high incidence of hyperphosphatemia in MHD patients is attributed to imbalance between phosphate intake and clearance, as dialysis remains the main route for removing excessive phosphate in patients with minimal kidney function^3^. Therefore, restriction of dietary phosphate intake in conjunction with the use of phosphate binders, is an important aspect of therapy for hyperphosphatemia^4^. However, restricting dietary phosphate often leads to a concomitant reduction of protein-rich foods intake, which inadvertently compromises dietary protein adequacy, thus increasing risk for protein energy wasting^5^.

There is a growing awareness in the variability of dietary phosphate bioavailability, both by source (plant or animal) and type (organic or inorganic). As such, bioavailability is lowest for phosphate derived from plants (10–30%), followed by phosphate derived from animals (40–60%), while it is highest (100%) for inorganic phosphates found in food additives^6^. The phosphate derived from plants is not readily digested and absorbed, as humans lack the necessary enzyme phytase to hydrolyse phytate to release phosphate. On the other hand, inorganic phosphates are salts that readily disassociate in the stomach, and as a result > 90% are absorbed^7^. Based on this understanding, the current approach in patient education should focus on choosing fresh food that contains phosphate with lower bioavailability, whereas avoiding food products with phosphate-containing additives^4,8^. It is also known that potential nutrient interactions in the digestive tract can affect dietary phosphate bioavailability^9^.

Dietary pattern is defined by the quantity, variety, or combination of different foods and beverages in a diet, and the frequency with which they are habitually consumed^10^. Dietary pattern analysis has emerged as a valuable approach to investigate associations between overall diet and health outcomes in human nutrition studies. In contrast to a singular nutrient/food-based approach, the dietary pattern-based approach takes into account that human food consumption falls into patterns reflecting a complex combination of dietary components and nutrients that are likely to have synergistic and competitive interactions^11^.

There are two approaches in dietary pattern analysis, namely *à priori* and *à posteriori*. An *à priori* approach evaluates the overall diet quality using scores or indices based on guidelines for a healthy diet or diets known to confer health benefits, whilst an *à posteriori* approach uses statistical methods to identify existing dietary patterns within a study population^12^. The *à posteriori* approach generates information on the population-specific dietary patterns, which facilitates a better understanding of health outcomes associated with locally relevant dietary patterns and development of culturally tailored nutrition intervention^13^. Appropriately, some studies have examined the association between the *à posteriori *derived dietary patterns and health related outcomes in MHD patients, such as nutritional risk^14^ and mortality^15,16^. A controlled feeding study in a non-chronic kidney disease (CKD) population has also reported that dietary phosphate bioavailability assessed using urinary phosphate excretion varies by dietary patterns^17^.

It is clear that the possible association between dietary patterns and serum phosphorus level in MHD patients remains unknown. Therefore, this study aimed to investigate the association between *à posteriori* derived dietary pattern and serum phosphorus level in MHD patients.

## Methods

### Study design and population

This was a cross-sectional study, which utilized data collected between October 2015 and November 2018 during screening of MHD patients for recruitment into the Palm Tocotrienols in Chronic Haemodialysis study, as described elsewhere^14,18^. Patients were recruited from 14 dialysis centres within the urban area of Klang Valley, Malaysia. Inclusion criteria included MHD patients aged at least 18 years old and dialyzed for at least 3 months. Exclusion criteria were patients with poor adherence toward haemodialysis treatment, unfit for assessment due to physical or mental disability, or with terminal illness such as HIV/AIDS or malignancy. Ethical approval was obtained from the Medical Research and Ethics Committee, Ministry of Health, Malaysia (reference number: NMRR-15-865-25260). All eligible patients gave written informed consent and all research procedures were conducted in accordance with relevant guidelines and regulations.

### Variables and data collection

Patients’ sociodemographic data, medical history, and the most recent drug prescription were retrieved from medical records. Patients’ self-reported compliance to phosphate binder prescriptions was assessed via face-to-face interviews. Biochemical results were extracted from in-centre patient laboratory reports relevant to within 2 weeks of collection of dietary data information. All analyses were performed by accredited laboratories^19^ in accordance with the operating procedures mandated by the Ministry of Health, Malaysia.

Dietary assessment was performed by research dieticians using the 3-day dietary recall (3-DDR) method, which included a dialysis day, a non-dialysis day, and a weekend day^20^. Common household measurement tools (bowls, spoons, and glasses) were used to optimize the portion size estimation. In addition, patients were asked about their weekly frequency of eating out. Food and beverages consumed in household units were transformed into absolute weight (g) and volume (ml) before analysis for nutrient composition using the Nutritionist Pro Software (First DataBank Inc., USA), which references the Malaysian Food Composition^21^ and Singapore Food Composition^22^ databases. The Goldberg’s index was used to identify 3DDRs of acceptable reporters to ensure the quality of dietary data. In brief, patients’ basal metabolic rate (BMR) was estimated using the Harris–Benedict equation^23^. Based on the reported energy intake (EI), EI:BMR ratios of < 1.2, 1.2–2.4, and > 2.4 were considered as under-, acceptable-, and over-reporting of 3DDRs respectively^24^.

Food items from the 3DDRs were classified into either animal or plant protein categories^25^. The animal protein group consisted of the following food items: fish, shellfish, eggs, poultry, red meat, milk, dairy products, processed or preserved meat, seafood, and eggs. The food sources of plant protein group were rice, cereals, beans, legumes, fruits, leafy vegetables, and starchy vegetables. An individual food item was directly assigned to the respective food group. For cooked dishes with a mixture of ingredients consisting of animal and plant proteins, recipes from the Malaysian Food Composition^21^ were referenced to determine the protein content of each ingredient, which was then assigned to the corresponding protein group.

The total dietary phosphate intake derived from the nutrient composition analysis of 3DDRs was categorized into organic phosphates (plant and animal foods), or inorganic phosphates (processed food or beverages). This categorization was based on the assumption that organic phosphate is naturally found in food, while inorganic phosphate is an additive in processed foods^26^. Accordingly, all food items from 3DDRs were divided into two food groups according to the source of phosphate. Food groups as the source of organic phosphate included cooked rice, soda crackers, fresh or frozen vegetables and fruits, eggs, beans, legumes, nuts, milk, fresh or frozen poultry, seafood, meats, and plain tea or coffee. The phosphate content of these food groups was further subdivided into either organic phosphate from plant or animal, based on the protein category as mentioned previously. Food groups as sources of inorganic phosphate were processed cheese, frozen meals, ready-to-eat cereals, cookies, canned, processed, and luncheon meat, poultry or seafood, canned soups, fast food, cola beverages, and beverages added with sweetened condensed milk. As these food groups might contain a combination of organic and inorganic phosphates, an assumption model was used to derive the added inorganic phosphate based on the difference of total phosphate content and protein content of foods in the unprocessed form. For composite dishes, standard recipes were referred to determine the content and source of phosphate of each constituent ingredient, which was then assigned to the corresponding group.

As the 3DDR method was chosen to assess dietary intake instead of a food frequency questionnaire, aggregation of food items into food groups was first carried out as previously described^14^ prior to performing the dietary pattern analysis. All food items from 3DDRs were extracted and sorted by alphabetical order. Then, duplicates were removed, and the food items were grouped based on similarity, food preparation method, and nutrient content. Initially, 47 food groups were developed and then, based on the consumption of each food group, food groups with consumption by less than 5% of patients were either excluded or collapsed with similar food groups. This narrowed the final food listing to 27 food groups.

The Diet Monotony Index (DMI) was calculated as described by Zimmerer et al.^27^ to assess food variety. From the 27-food group listing developed, the total quantity of each food group consumed was converted into servings according to the Malaysian Dietary Guideline 2010^28^. A diet consumed with a wide variety of food groups resulted in smaller proportions of total servings for each food group, and a smaller index value was derived, or vice versa.

### Statistical analyses

Continuous variables were presented as mean ± SD or median [interquartile range (IQR)], while categorical variables were presented as frequency (percentages). Non-normally distributed variables were log-transformed before statistical analyses. *Chi-square* test was used to determine associations between two categorical variables. Simple linear regression was used to determine the correlation between serum phosphorus (dependent variable) and clinical and dietary parameters (independent variables). Variables with *p*-value < 0.1 on univariate analysis were included in the subsequently multiple linear regression analysis. *Tolerance* and *variance inflation factor* were used to check for multicollinearity.

Dietary patterns were derived using *à posteriori* approach as previously described^14^. The input variable for factor analysis was the weight of each food group. Principal component analysis (PCA) was used to derive dietary patterns and the derived patterns were orthogonally rotated (varimax rotation) to enhance the difference between loadings to improve the interpretability of factors. The number of dietary patterns retained was determined based on eigenvalue > 2.0^29^, scree plot examination, and interpretability of the derived patterns^30^. The eigenvalue indicates the total variance explained by a given factor^30^. Dietary patterns were named in accordance to the food group with the highest factor loading. Patients were assigned to factor scores computed for each pattern identified, which indicated adherence to that pattern. Based on the factor score, patients were categorized into tertiles (T1–T3) for each dietary pattern, where tertile 1 (T1) represented the lowest adherence while tertile 3 (T3) was the highest adherence to that pattern.

One-way ANOVA was used to compare nutrient intakes by tertiles of identified dietary patterns, with Bonferroni test for *post hoc * analyses. Kruskal–Wallis with Dunn’s *post hoc* test was used for comparison of non-normally distributed variables. One-way analysis of covariance adjusted for covariates was used to compare serum phosphorus by tertiles of each dietary pattern, and Bonferroni test was used for multiple comparisons. Multiple logistic regressions were used to estimate the odds ratios (ORs) of hyperphosphatemia (serum phosphorus above 1.78 mmol/l and 2.00 mmol/l) associated with T3 of each dietary pattern. Missing covariate data (less than 2%) were imputed using the mean of the existing value of all patients. All analyses were computed using the SPSS version 25 (IBM, Chicago, IL, USA). Statistical significance was set at *p*-value < 0.05 for all evaluated parameters.

## Results

**Figure 1:**
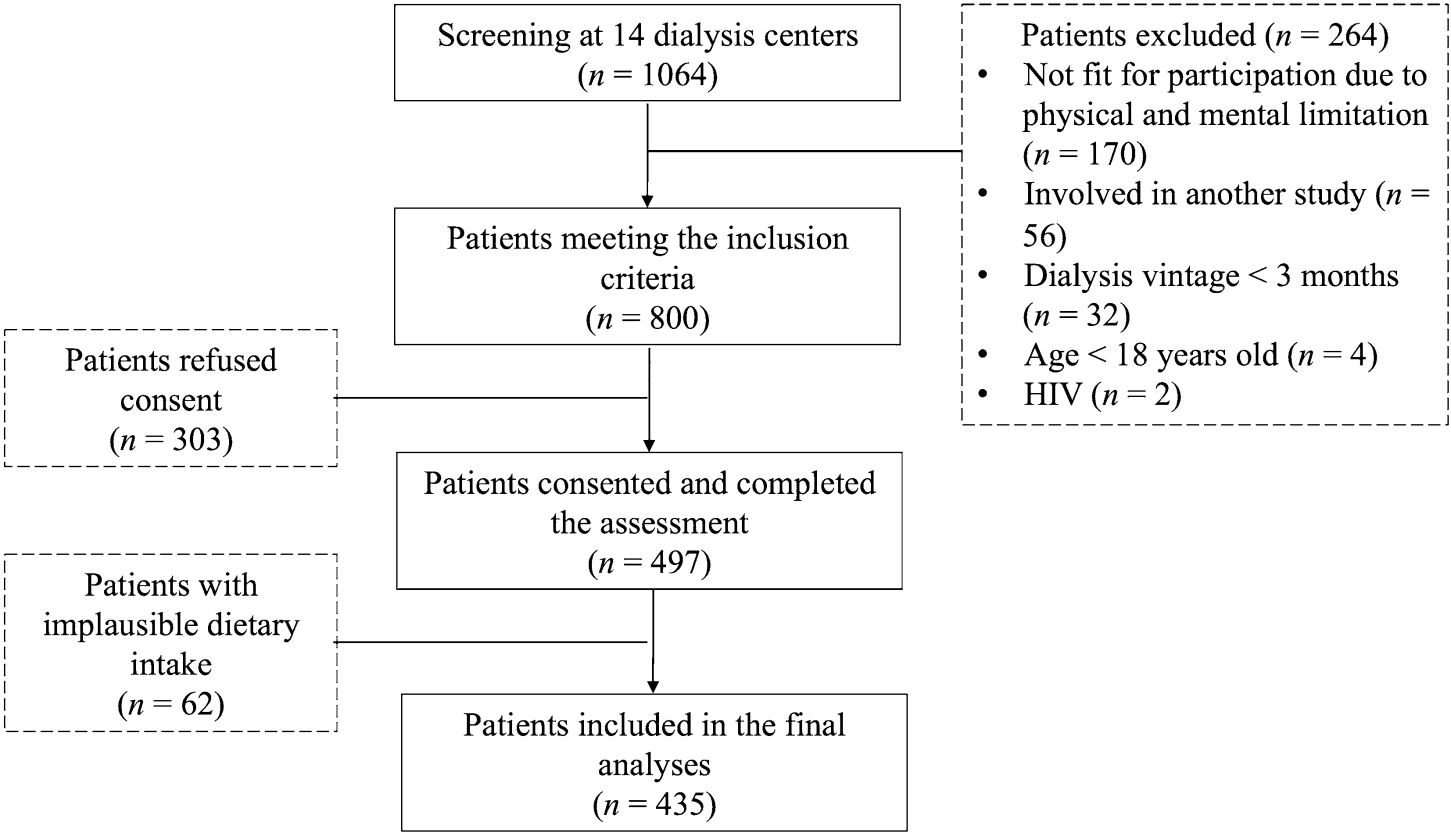
Study flow chart for patient recruitment.

The flow of patient enrolment is shown in Fig. 1. Of 800 eligible patients, 497 consented and completed all assessments. The 303 eligible patients who refused consent were reluctant to commit time for research procedures, as they were exposed to many research study in their settings.  In addition, they were also experiencing fatigue when reporting dietary data. Out of 497 patients who completed the assessment, 62 (12.5%) were excluded due to implausible dietary data. Therefore, the final data analyses included 435 MHD patients, whose characteristics are shown in Table 1. This was a multiethnic population with Malay (42.3%), Chinese (39.8%), and Indian (17.9%) patients, with a mean age of 55 ± 13 years, median dialysis vintage of 56 months, and with 54.8% males. All patients were dialyzed thrice weekly for four hours per session as per the clinical practice guideline by the Ministry of Health, Malaysia^31^. Majority of the patients were recruited from dialysis centres of non-governmental organizations (46.4%). The mean serum phosphorus was 1.78 ± 0.50 mmol/l and 47.8% patients had serum phosphorus above 1.78 mmol/l. Patients with serum phosphorus level above 1.78 mmol/l were mainly from non-governmental dialysis centres (*p* = 0.024) and did not comply to using phosphate binder (*p* = 0.002) (Supplementary Table S1). Phosphate binders were prescribed to 98.2% patients and the type of phosphate binder was mainly calcium-based (92.9%). However, only 59.5% reported adhering to the phosphate binder prescription. The median frequency of eating out weekly was 8 (IQR = 10).

**Table 1:** Characteristics of patients (n = 435).

Characteristics	Mean ± SD	*n* (%)
Age (year)	54.6 ± 13.4	
**Sex**		
Male		239 (54.9)
Female		196 (45.1)
**Ethnicity**		
Malay		184 (42.3)
Chinese		173 (39.8)
Indian		78 (17.9)
**Sector of dialysis provider**		
Government		165 (37.9)
Non-governmental organization		202 (46.5)
Private		68 (15.6)
**Anthropometry**		
Weight (kg)	62.5 ± 14.2	
Height (cm)	158.1 ± 8.8	
Body mass index (kg/m^2^)	24.9 ± 5.0	
**Biochemistry**		
Pre-dialysis urea (mmol/l)	19.2 ± 5.4	
Pre-dialysis creatinine (μmol/l)	820 (271)*	
Serum potassium (mmol/l)	5.0 ± 0.8	
Serum phosphorus (mmol/l)	1.78 ± 0.50	
<1.18 mmol/l		47 (10.8)
1.18–1.78 mmol/l		180 (41.4)
> 1.78 mmol/l		208 (47.8)
Serum corrected calcium (mmol/l)	2.26 ± 0.24	
Serum alkaline phosphatase (IU/l)	103 (76)*	
Serum albumin (g/l)	39.3 ± 4.0	
**Medical background**		
Co-morbidities		
Diabetes mellitus		190 (43.7)
Hypertension		349 (80.2)
Hepatitis B/C		47 (10.8)
Dialysis vintage (month)	56 (73)*	
Kt/V	1.6 ± 0.4	
nPNA (g/kg)	1.0 ± 0.3	
Parathyroidectomy history		69 (15.9)
**Medication prescriptions**		
Phosphate binder prescription		427 (98.2)
Calcium based phosphate binder		404 (92.9)
Lanthanum carbonate		17 (3.9)
Sevelamer carbonate		6 (1.4)
Activated vitamin D prescription		299 (68.7)
Self-reported adherence to phosphate binder		254 (59.5)
**Dietary intake**		
Energy (kcal)	1524 ± 343	
Energy (kcal/kg)	25.2 ± 6.5	
Total protein (g)	52.5 (20.6)*	
Total protein (g/kg)	0.9 (0.4)*	
Animal protein (g)	28.2 (19.5)*	
Plant protein (g)	23.7 (10.0)*	
Total phosphate (mg)	634.8 (268.0)*	
Animal organic phosphate (mg)	236.6 (206.0)*	
Plant organic phosphate (mg)	269.7 (160.0)*	
Inorganic phosphate (mg)	80.7 (137.0)*	
Phosphate to protein ratio (mg/g)	12.0 (3.4)*	
Total fluid (ml)	1246 (830)*	
Eating out frequency (per week)	8 (10)*	
Diet Monotony Index	30 (12)*	

nPNA normalization of protein nitrogen appearance.

*Data is presented as median with interquartile range (IQR).

**Figure 2:**
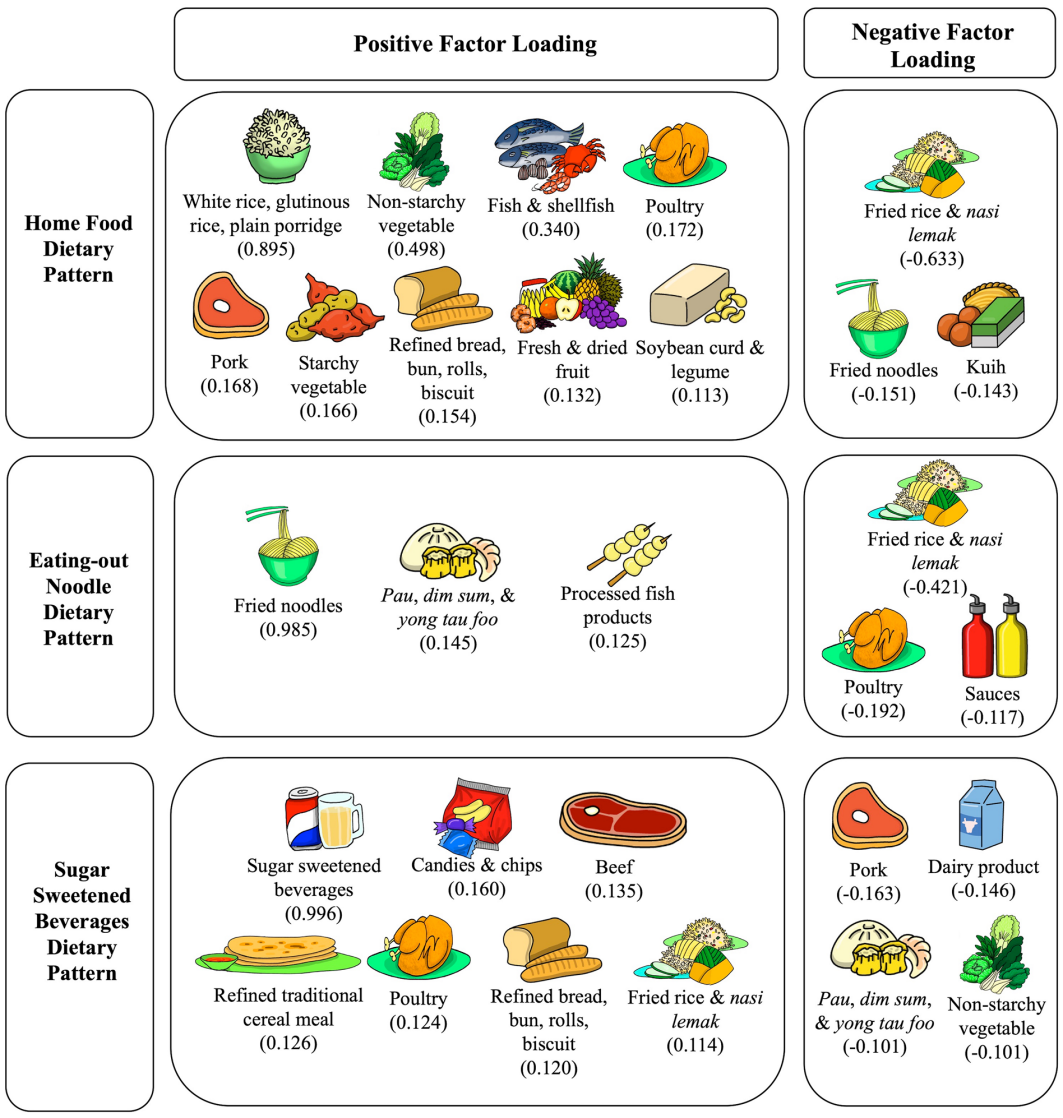
Food groups with factor loading within each dietary pattern. The factor loading indicates correlations of the food group with the dietary pattern. Local ethnic-based food names are *Kuih*—local sweet or savoury bite sized snacks; *Pau*—filled steamed bun; *Dim Sum*—bite-sized dumpling filled with meat or seafood; *Yong Tau Hoo*—soybean curd or vegetables filled with fish paste or ground meat; *Nasi Lemak—*rice cooked in coconut milk.

Three dietary patterns emerged with a total of 27 food groups using PCA (Table 2). The Kaiser–Meyer–Olkin value of PCA was 0.538, indicating acceptable sampling adequacy for factor analysis. The first dietary pattern was labelled as “Home Food_DP_” as it represented a high intake of white rice, non-starchy vegetables, fish and shellfish, poultry, pork, refined bread, bun, roll, and biscuit, fresh and dried fruit, and soybean curd and legume, along with a low intake of fried rice and coconut milk rice (Fig. 2). The second dietary pattern was labelled as “Eating-out Noodles_DP_” as it reflected a high intake of fried noodle dishes, *pau*, *dim sum* and *yong tau foo*, and processed fish products, along with a low intake of fried rice and *nasi lemak*. The third dietary pattern was characterized by a high intake of sugar-sweetened beverages, along with candies and chips, beef, refined traditional cereal meal, poultry, refined bread, bun, roll and biscuit, and fried rice and *nasi lemak*. This pattern was labelled as “Sugar-Sweetened Beverages_DP_ (SSB_DP_)”. These three dietary patterns explained 72.1% of the total variance among 27 food groups, with the SSB_DP_ accounting for the highest variance (45.3%), followed by Home Food_DP_ (13.8%), and Eating-out Noodles_DP_ (13.0%) patterns.

**Table 2:** Factor loadings for three dietary patterns derived by principal component analysis.

Food groups	Components		
	Home food	Eating out noodles	Sugar sweetened beverages
Beef	− 0.022	− 0.044	**0.135**
Candies and chips	− 0.013	− 0.010	**0.160**
Chicken egg	0.093	− 0.038	0.025
Dairy products	− 0.021	− 0.027	− **0.146**
Fish and shellfish	**0.340**	0.011	− 0.027
Fresh and dried fruit	**0.132**	− 0.061	− 0.092
Fried rice and *nasi lemak*	− **0.633**	− **0.421**	**0.114**
*Kuih*	− **0.143**	− 0.088	0.003
Non-starchy vegetables	**0.498**	− 0.053	− **0.101**
Noodles, fried	− **0.151**	**0.958**	− 0.010
Noodles, soup	0.079	0.056	− 0.026
*Pau*, *dim sum* and *yong tau foo*	− 0.046	**0.145**	− **0.110**
Pork	**0.168**	0.058	− **0.163**
Poultry	**0.172**	− **0.192**	**0.124**
Preserved fish, shell fish, poultry, egg, and meat	0.067	− 0.001	0.016
Preserved vegetables	0.048	0.074	− 0.059
Processed chicken and red meat products	− 0.023	0.003	− 0.030
Processed fish products	− 0.008	**0.125**	0.053
Refined bread, bun, roll and biscuit	**0.154**	− 0.079	**0.120**
Refined traditional cereal meal	0.039	− 0.083	**0.126**
Sauces	− 0.023	− **0.117**	0.066
Soybean curd and legume	**0.113**	0.014	− 0.006
Spreads (fat)	0.077	− 0.024	0.008
Spreads (sweet)	− 0.027	− 0.010	0.076
Starchy vegetables	**0.166**	− 0.025	− 0.100
Sugar sweetened beverages	− 0.028	− 0.080	**0.996**
White rice, glutinuous rice, and plain rice porridge	**0.895**	− 0.043	0.074

Data expressed as factor loading (correlation coefficient between each food group and dietary pattern); foods groups with factor loadings ≥ 0.1 were bolded to indicate main food groups in each factor; principal component analysis with eigenvalue > 2.0 and orthogonal rotation for derivation of dietary patterns.

Local ethnic-based food names are *Kuih*—local sweet or savory bite sized snacks; *Pau*—filled steamed bun; *Dim Sum*—bite-sized dumpling filled with meat or seafood; *Yong Tau Hoo*—soybean curd or vegetables filled with fish paste or ground meat; *Nasi Lemak—*rice cooked in coconut milk.

The linear regression analyses of serum phosphorus are shown in Table 3. In the model accounting for all variables, dialysis adequacy (Kt/V, *β* = − 0.223, *p* = 0.001), adherence to phosphate binders (*β* = − 0.184, *p* < 0.001), BMI (*β* = 0.016, *p* = 0.001), and age (*β* = − 0.005, *p* = 0.009) were negatively correlated to serum phosphorus level, whilst normalization of protein nitrogen appearance (*β* = 0.242, *p* = 0.005) was positively correlated to serum phosphorus level.

**Table 3:** Factors associated with serum phosphate levels using linear regression analyses.

Variables	Simple linear regression		Multiple linear regression^†^	
	β (95% CI)	*P* value	adjusted β (95% CI)	*P* value
Age (year)	− 0.004 (− 0.008, − 0.001)	**0.016**	− 0.005 (− 0.008, − 0.001)	**0.009**
Sex (male)	0.071 (− 0.024, 0.165)	0.144	− 0.010 (− 0.110, 0.091)	0.852
BMI (kg/m^2^)	0.018 (0.009, 0.027)	**< 0.001**	0.016 (0.006, 0.026)	**0.001**
Dialysis vintage (month)*	0.018 (− 0.030, 0.065)	0.465	–	
Parathyroidectomy (yes)	− 0.006 (− 0.136, 0.123)	0.923	–	
Adherence to phosphate binder (yes)	− 0.158 (− 0.252, − 0.063)	**0.001**	− 0.184 (− 0.276, − 0.092)	**< 0.001**
Kt/V	− 0.214 (− 0.327, − 0.101)	**< 0.001**	− 0.223 (− 0.357, − 0.089)	**0.001**
*n*PNA (g/kg)	0.164 (0.000, 0.329)	**0.050**	0.242 (0.073, 0.411)	**0.005**
Total protein (g)*	0.205 (0.063, 0.346)	**0.005**	–	
Animal protein (g)*	0.031 (0.003, 0.059)	**0.029**	0.018 (− 0.034, 0.071)	0.498
Plant protein (g)*	0.052 (− 0.016, 0.121)	0.135	0.033 (− 0.034, 0.101)	0.331
Total phosphate (mg)*	0.124 (− 0.010, 0.258)	0.069	0.015 (− 0.126, 0.157)	0.830
Animal organic phosphate (mg)*	0.016 (− 0.004, 0.037)	0.122	− 0.005 (− 0.043, 0.033)	0.782
Plant organic phosphate (mg)*	0.007 (− 0.048, 0.062)	0.810	–	
Inorganic phosphate (mg)*	0.000 (− 0.015, 0.014)	0.946	–	
Phosphate to protein ratio (mg/g)*	− 0.131 (− 0.334, 0.072)	0.205	–	

*n*PNA, normalization of protein nitrogen appearance.

*Data was log-transformed.

“–” indicates variables not included in the multiple linear regression analysis; only variables with *p*-value < 0.25 in the simple linear regression were carried into the multiple linear regression analysis, “total protein” was excluded as it was a sum of both “animal protein” and “plant protein” while “phosphate to protein ratio” was excluded in the analysis due to collinearity. Bold values denote statistical significance at *p*-value < 0.05.

^†^The highest variance inflation factor was 3.960.

The nutrient profiles of three identified dietary patterns are presented in Table 4. The upper tertile (T3) of Home Food_DP_ indicated significantly higher intakes of total protein (*p* = 0.002), animal protein (*p* = 0.001), animal-based organic phosphate (*p* < 0.001), and total fluid (*p* = 0.003). On the other hand, the T3 of SSB_DP_ indicated significantly higher intakes of total energy (*p* < 0.001), inorganic phosphate (*p* < 0.001), phosphate to protein ratio (*p* = 0.001), and total fluid (*p* = 0.010). The T3 of Eating-out Noodles_DP_ indicated significantly greater intakes of total energy (*p* = 0.033), total protein (*p* = 0.003), plant protein (*p* < 0.001) but lower phosphate to protein ratio (*p* = 0.009). Of note, the frequency of eating out was significantly greater in T3 of SSB_DP_ (*p* = 0.002) and Eating-out Noodles_DP_ (*p* = 0.009). Patients of T3 of Eating-out noodle_DP_ were mostly Chinese and from non-governmental dialysis centers while patients of T3 of SSB_DP_ were younger, consisted of mostly men and Malay, and from governmental dialysis centers (Supplementary Table S2). The Home Food_DP_ was not associated with age, gender, ethnic, and sector of dialysis provider.

**Table 4:** Comparison of dietary parameters between tertiles for each dietary pattern.

	Home Food_DP_			*P* trend	*P* value* _T1vsT3_
	Tertile 1 (*n *= 142)	Tertile 2 (*n *= 144)	Tertile 3 (*n *= 149)		
Energy (kcal)	1490 ± 359	1564 ± 366	1519 ± 301	0.183	> 0.999
Energy (kcal/kg)	24.6 ± 6.0	25.9 ± 7.4	25.0 ± 5.9	0.244	> 0.999
Total protein (g)	48.7 (23.0)	52.6 (21.9)	54.2 (17.9)	**0.002**	**0.002**
Total protein (g/kg)	0.8 (0.5)	0.8 (0.4)	0.9 (0.4)	**0.010**	**0.007**
Animal protein (g)	24.0 (22.0)	26.7 (19.6)	32.1 (16.7)	**0.001**	**0.001**
Plant protein (g)	22.7 (11.2)	23.9 (9.2)	24.8 (9.6)	0.474	–
Total phosphate (mg)	625 (284)	632 (328)	648 (237)	0.206	–
Animal organic phosphate (mg)	196 (201)	251 (186)	282 (198)	**< 0.001**	**< 0.001**
Plant organic phosphate (mg)	262 (183)	273 (161)	274 (133)	0.247	–
Inorganic phosphate (mg)	89 (147)	91 (148)	67 (108)	**0.026**	0.055
Phosphate to protein ratio (mg/g)	12.1 (4.1)	12.2 (3.3)	11.9 (2.9)	0.249	–
Total fluid (ml)	1177 (730)	1258 (966)	1336 (776)	**0.004**	**0.003**
Dietary monotony index	30 (13)	30 (11)	29 (13)	0.803	–
Eating out frequency (per week)	8 (10)	9 (11)	7 (10)	**0.027**	> 0.999

	Sugar Sweetened Beverages_DP_			*P* trend	*P* value* _T1vsT3_
	Tertile 1 (*n *= 145)	Tertile 2 (*n *= 145)	Tertile 3 (*n *= 145)		
Energy (kcal)	1431 ± 334	1491 ± 332	1651 ± 328	**< 0.001**	**< 0.001**
Energy (kcal/kg)	23.5 ± 5.9	25.6 ± 6.8	26.5 ± 6.4	**< 0.001**	**< 0.001**
Total protein (g)	53.3 (20.6)	51.5 (22.3)	51.1 (21.2)	0.583	–
Total protein (g/kg)	0.9 (0.4)	0.9 (0.5)	0.8 (0.4)	0.525	–
Animal protein (g)	29.9 (22.0)	27.7 (19.7)	27.4 (17.0)	0.411	–
Plant protein (g)	22.7 (10.4)	24.4 (9.6)	24.3 (11.2)	0.233	–
Total phosphate (mg)	604 (263)	639 (290)	683 (278)	0.168	–
Animal organic phosphate (mg)	230 (230)	242 (217)	237 (195)	0.670	–
Plant organic phosphate (mg)	264 (163)	274 (156)	270 (157)	0.595	–
Inorganic phosphate (mg)	63 (106)	64 (116)	128 (167)	**< 0.001**	**< 0.001**
Phosphate to protein ratio (mg/g)	11.4 (3.5)	12.0 (3.1)	12.7 (3.2)	**0.001**	**0.001**
Total fluid (ml)	1183 (1032)	1173 (632)	1350 (795)	**< 0.001**	**0.010**
Dietary monotony index	28 (10)	30 (15)	30 (10)	0.411	–
Eating out frequency (per week)	7 (10)	7 (10)	10 (9)	**0.002**	**0.002**

	Eating-out Noodle_DP_			*P* trend	*P* value* _T1vsT3_
	T1 (*n *= 202)	T2 (*n *= 116)	T3 (*n *= 117)		
Energy (kcal)	1491 ± 360	1549 ± 323	1557 ± 331	0.166	0.293
Energy (kcal/kg)	23.9 ± 6.7	26.6 ± 6.4	25.8 ± 5.9	**0.001**	**0.033**
Total protein (g)	49.1 (20.1)	53.9 (15.6)	53.1 (27.2)	**0.016**	0.062
Total protein (g/kg)	0.8 (0.3)	0.9 (0.4)	1.0 (0.5)	**< 0.001**	**0.003**
Animal protein (g)	26.9 (18.8)	30.0 (17.4)	28.0 (24.4)	0.476	–
Plant protein (g)	22.1 (9.9)	23.2 (10.6)	26.1 (9.1)	**< 0.001**	**< 0.001**
Total phosphate (mg)	634 (264)	634 (257)	638 (327)	0.847	–
Animal organic phosphate (mg)	238 (215)	248 (167)	226 (241)	0.517	–
Plant organic phosphate (mg)	253 (169)	273 (169)	289 (150)	0.390	–
Inorganic phosphate (mg)	77 (149)	85 (151)	79 (106)	0.725	–
Phosphate to protein ratio (mg/g)	12.4 (3.6)	12.1 (2.9)	11.4 (3.1)	**0.004**	**0.003**
Total fluid (ml)	1347 (1007)	1209 (833)	1222 (571)	0.207	–
Dietary monotony index	30 (11)	28 (9)	31 (13)	**0.044**	0.851
Eating out frequency (per week)	8 (10)	8 (9)	9 (10)	**0.011**	**0.009**

Data is expressed as mean ± standard deviation or median with interquartile range (IQR). Bold values denote statistical significance at p-value < 0.05.

*Significance values had been adjusted for multiple tests.

“–” The Dunn’s post hoc test was not performed if the overall test was not significant.

*T1* tertile 1, *T2* tertile 2, *T3* tertile 3.

**Figure 3:**
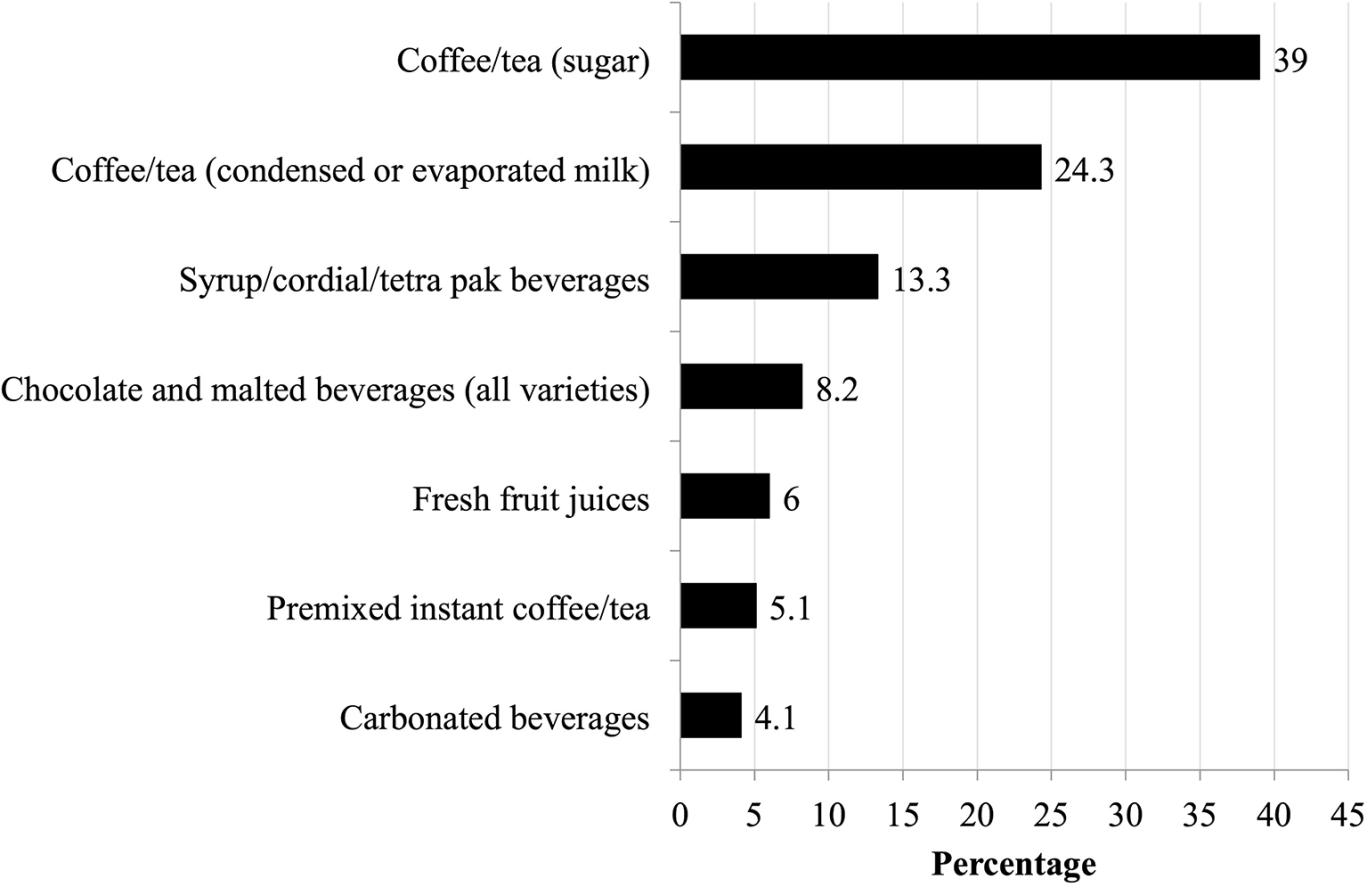
Types of beverages consumed by tertile 3 patients (*n* = 145) within the sugar sweetened beverages dietary pattern.

Serum phosphorus level by tertiles for each dietary pattern is presented in Table 5. After adjustment for confounders, serum phosphorus levels were found to be significantly higher for T3 vs T1 of SSB_DP_ (*p* = 0.006). Although, no T3 of any dietary pattern significantly was associated with a higher OR for serum phosphorus level > 1.78 mmol/l (*p* > 0.05), still a non-significant trend (*p* = 0.067) was observed with the SSB_DP_ (OR = 1.64; 95% CI 0.97, 2.79), which could be considered significant for *p* value < 0.10. We found, however, that the OR for elevating serum phosphorous > 2.00 mmol/l, a cut-off that is clinically relevant, significantly increased by 2.35 times (95% CI 1.30, 4.28, *p* = 0.005) with T3 of SSB_DP_. Coffee or tea added with sugar was the most common SSB (39.0%) consumed by patients in T3 of SSB_DP_, followed by coffee or tea added with condensed milk or evaporated milk (24.3%), and syrup or cordial or tetra pak beverages (13.3%) (Fig. 3).

**Table 5:** Serum phosphorus and odds ratio of hyperphosphatemia (serum phosphorus > 1.78 mmol/l and 2.00 mmol/l) by tertiles of dietary patterns.

Dietary pattern	Serum phosphorus (mmol/l)*		Serum phosphorus > 1.78 mmol/l^†^		Serum phosphorus > 2.00 mmol/l^†^	
	Model 1	Model 2	Model 1	Model 2	Model 1	Model 2
**Home food**						
Tertile 1	1.76 ± 0.04	1.80 ± 0.05	1.00	1.00	1.00	1.00
Tertile 2	1.72 ±0.05	1.77 ± 0.05	0.68 (0.42, 1.09)	0.67 (0.41, 1.09)	0.78 (0.46, 1.33)	0.81 (047, 1.39)
Tertile 3	1.74 ± 0.04	1.78 ± 0.05	0.70 (0.43, 1.13)	0.69 (0.42, 1.13)	0.91 (0.54, 1.54)	0.87 (0.51, 1.50)
*P* trend	0.829	0.830	0.208	0.119	0.661	0.737
*P* value for T1 vs T3	> 0.999	> 0.999	0.141	0.137	0.735	0.622
**Sugar sweetened beverages**						
Tertile 1	1.67 ± 0.05	1.70 ± 0.05	1.00	1.00	1.00	1.00
Tertile 2	1.71 ± 0.04	1.75 ± 0.05	1.00 (0.61, 1.62)	1.03 (0.62, 1.71)	1.12 (0.64, 1.96)	1.29 (0.72, 2.31)
Tertile 3	1.85 ± 0.05	1.88 ± 0.05	1.61 (0.97, 2.67)	1.64 (0.97, 2.79)	1.99 (1.14, 3.48)	2.35 (1.30, 4.28)
*P* trend	**0.008**	**0.005**	0.097	0.108	**0.030**	**0.013**
*P* value for T1 vs T3	**0.011**	**0.006**	0.066	0.067	**0.015**	**0.005**
**Eating out noodles**						
Tertile 1	1.73 ± 0.04	1.77 ± 0.04	1.00	1.00	1.00	1.00
Tertile 2	1.74 ± 0.05	1.78 ± 0.06	1.04 (0.64, 1.71)	1.04 (0.63, 1.72)	1.14 (0.66, 1.96)	1.20 (0.69, 2.09)
Tertile 3	1.77 ± 0.06	1.82 ± 0.06	1.00 (0.60, 1.66)	1.03 (0.61, 1.73)	1.10 (0.63, 1.92)	1.18 (0.66, 2.11)
*P* trend	0.868	0.745	0.985	0.987	0.881	0.756
*P* value for T1 vs T3	> 0.999	> 0.999	0.993	0.918	0.744	0.568

Model 1 is adjusted for age, gender, ethnic, sector of dialysis provider; Model 2 is adjusted for all confounders in model 1 and dialysis vintage, Kt/V, normalization of protein nitrogen appearance, history of parathyroidectomy, prescription of activated vitamin D, and self-reported compliance to phosphate binder.

*Data is presented as mean ± standard error, ^†^data is presented as odds ratio (95% confidence interval). Bold values denote statistical significance at p-value < 0.05.

A total of 105 patients of T3 of Home Food_DP_ and 101 patients of T3 of SSB_DP_ were identified and comparison between these two groups was made (Table 6). The T3 patients of SSB_DP_ were younger (*p* = 0.001); mainly men (*p* = 0.037) and likely Malay patients (*p* < 0.011) compared to T3 patients of Home Food_DP_. The total energy intake (*p* < 0.011), inorganic phosphate intake (*p* < 0.011) and phosphate to protein ratio (*p* = 0.001) were significantly higher while total protein intake (*p* = 0.044), animal protein intake (*p* = 0.004), and organic phosphate from animal sources (*p* = 0.003) were significantly lower in T3 patients of SSB_DP_ compared to T3 patients of Home Food_DP_. The serum phosphorus level of T3 patients of SSB_DP_ was significantly greater than T3 patients of Home Food_DP_ (1.87 ± 0.49 vs*.* 1.71 ± 0.47 mmol/l, *p* = 0.021), and remained significant (*p* = 0.014) after adjustment for confounding factors.

**Table 6:** Comparison of characteristics between patients of tertile 3 of home food and sugar sweetened beverages dietary patterns.

	Tertile 3 Home Food_DP_ (*n* = 105)	Tertile 3 SSB_DP_ (*n* = 101)	*P* value
Age (year)	58.6 ± 12.6	52.6 ± 12.1	**0.001**
**Gender**			
Male	58 (55.2%)	70 (69.3%)	**0.037**
Female	47 (44.8%)	31 (30.7%)	
**Sector**			
Government	36 (34.3%)	45 (44.6%)	0.050
NGO	54 (51.4%)	35 (34.7%)	
Private	15 (14.3%)	21 (20.8%)	
**Ethnic**			
Malay	34 (32.4%)	59 (58.4%)	**< 0.001**
Chinese	54 (51.4%)	26 (25.7%)	
Indian	17 (16.2%)	16 (15.8%)	
**Compliance to binder**			
Yes	39 (37.5%)	42 (42.9%)	0.437
No	65 (62.5%)	56 (57.1%)	
Kt/V	1.66 ± 0.43	1.65 ± 0.27	0.909
Energy (kcal/kg)	1456 ± 266	1644 ± 327	**< 0.001**
Protein (g/kg)*	54.1 (17.4)	49.5 (20.2)	**0.044**
Phosphate (mg)*	607 (228)	632 (280)	0.584
Plant protein (g)*	24.4 (8.6)	23.5 (9.5)	0.688
Animal protein (g)*	31.9 (18.6)	24.7 (16.4)	**0.004**
Organic plant phosphate (mg)*	267 (131)	265 (164)	0.353
Organic animal phosphate (mg)*	283 (199)	231 (186)	**0.003**
Inorganic phosphate (mg)*	45 (98)	147 (158)	**< 0.001**
Phosphate protein ratio*	11.6 (3.1)	12.9 (3.6)	**0.001**
Serum phosphorus (mmol/l)	1.71 ± 0.47	1.87 ± 0.49	**0.021**
	1.75 ± 0.06^†^	1.92 ± 0.06^†^	**0.014**^†^
**Category of serum phosphorus**			
≤ 1.78 mmol/l	61 (58.1%)	45 (44.6%)	0.052
> 1.78 mmol/l	44 (41.9%)	56 (55.4%)	

Continuous data was presented as mean ± SD and independent *t* test was used for analyses while categorical data was presented as frequency (%) and chi-square test was used for analysis.

*DP* dietary pattern, *NGO* non-governmental organization, *SSB* sugar sweetened beverages.

*Data was presented as median with interquartile range (IQR) and Mann–Whitney test was used for analyses. Bold values denote statistical significance at p-value < 0.05.

^†^Data was presented as mean ± SE and the general linear model was used for analysis with adjustment for age, gender, ethnic, sector of dialysis provider, dialysis vintage, Kt/V, normalization of protein nitrogen appearance, history of parathyroidectomy, prescription of activated vitamin D, and self-reported compliance to phosphate binder.

Forty-four patients were excluded in these analyses as they were categorized into tertile 3 of both Home Food_DP_ and SSB_DP_.

## Discussion

In this cross-sectional study of 435 MHD patients, three dietary patterns emerged through the *à posteriori* approach, namely Home Food_DP_, SSB_DP_, and Eating-out Noodle_DP_. The empirically derived dietary patterns reflected the habitual dietary intake within this MHD population. Similar dietary patterns were also reported in an earlier study with a smaller population^14^. In the data reported by Sulaheen et al.^14^, the first 382 patients enrolled were used in the primary analyses, which focused on malnutrition. In the present study, we assessed the subsequent 497 enrolled patients for hyperphosphatemia risk. However, dietary patterns reported were different due to sample size, ethnicity proportion, and analyses. In the study by Sualeheen et al.^14^, four dietary patterns were identified, namely Home Food, Eating-out Noodle, Eating-out Rice, and Eating-out SSB. The dietary pattern with the highest variance was Eating-out Noodle (17.0%). Contrarily, we used eigenvalue > 2.0 in the PCA for the present study, which led to retention of only three dietary patterns, with the SSB_DP_ accounting for the highest variance (45.3%). It should be also noted that the patients in the study by Sualeheen et al.^14^ were mainly Chinese (45%), followed by Malay (36%) and Indian (19%) whilst an almost equal number of Malay and Chinese patients were recruited in the present study. The trend of studies examining dietary patterns for CKD populations does note that these patterns reflect local food habits. The DIET-HD study identified “fruits and vegetables” and “Western” dietary patterns for 8,110 MHD patients across 10 European countries and Argentina^16^. These dietary patterns are also consistent with reported patterns for the general population within these same countries^32^. The *Reasons for Geographic and Racial Differences in Stroke* study conducted for CKD populations in the South-eastern United States, identified a “Southern” dietary pattern characterized by fried foods, organ meats, and sweetened beverages, which is typical of Southern cuisine^33^. Similarly, the PROGREDIR study conducted among pre-dialysis CKD patients in São Paulo, Brazil identified a “traditional” dietary pattern (composed of white rice, beans, and coffee) typical to the local community^34^.

In this study, we did not observe any significant association between serum phosphorus level and any individual nutrient, including dietary phosphate. There are some possible explanations for this null finding. Firstly, total dietary phosphate intake does not reflect the actual amount of phosphate absorbed in the intestinal tract, as phosphate bioavailability varies by food source^7^. Secondly, patients’ dietary phosphate intakes were estimated from 3DDRs with reference to food composition databases. Other studies have indicated discrepancies in phosphate content estimated from diet recalls using food composition databases with direct laboratory analyses of duplicate portions of diet samples^35,36^. The wide use of phosphate additives in food manufacturing adds to actual burden of dietary phosphate intake as opposed to the lower estimations from referencing the food composition databases^37^. In addition, we observed variables such as dialysis adequacy (Kt/V) and adherence to phosphate binder prescription were inversely associated with serum phosphorus level. This implies that management of hyperphosphatemia in MHD patients is beyond limiting total amount of dietary phosphate intake alone. In fact, Lynch et al.^38^ have shown that prescribed dietary phosphate restrictions could not be associated with improving survival. In the present study, more patients from non-governmental dialysis centers had serum phosphorus level above 1.78 mmol/l compared to patients from government or private centers. This finding is consistent with our previous study, which reported the prevalence of hyperphosphatemia in MHD patients based on the data from Malaysian National Renal Registry^39^. A possible explanation is that government and private centers have more resources in terms of manpower and treatment options for achieving the target serum phosphorus level.

We went beyond the single nutrient approach and examined the effect of dietary patterns on serum phosphorus level. We found that the SSB_DP_ was significantly associated with higher serum phosphorus levels, which could be attributed to greater consumption of inorganic phosphate as found in beverages added with condensed milk. It is well known that cola beverages contain phosphoric acid additives that contribute to substantial dietary phosphate burden^40^. However, our study showed that carbonated beverages were the least consumed SSB. Instead, tea or coffee added with sugar, sweetened condensed milk or evaporated milk were the most popular choice of SSB in our population as indicated by national trade estimates data^41^. Inorganic phosphate additives such as sodium phosphates, calcium phosphates, triphosphates, and polyphosphates are added as stabilizing salts during the manufacturing process of evaporated and sweetened condensed milk which according to CODEX standards may be between 0.2 to 0.3% of product content^42,43^. We examined the front-on-pack labelling of popular evaporated and sweetened condensed milk brands in the market, but found none disclosed the phosphate content or type of additives. The non-mandatory reporting of phosphate content in food labelling has made it difficult for MHD patients to identify potential hidden sources of phosphate^44^. In the United States, a cross-sectional analysis from the National Health and Nutrition Examination Survey revealed that consumption of dairy products and cereals or grains with inorganic phosphate additives were also associated with greater serum phosphorus in the general population^26^. Contrarily, a recent dietary pattern study on African American patients on MHD did not observe significant difference in serum phosphorus level between high SSB_DP_ and low SBB_DP_, likely due to a smaller sample size, a homogenous population and different food selection^45^.

Although the Home Food_DP_ was associated with greater dietary protein intake, particularly animal protein, this pattern did not adversely affect serum phosphorus levels. Typical protein foods implicated in the Home Food_DP_ were fish and shellfish, poultry, pork, and soybean curd and legumes, which did not occur in the other two patterns. At the same time, the Home Food_DP_ also carried appreciable factor loadings for non-starchy vegetables (0.498), starchy vegetables (0.166), and fresh and dried fruit (0.132). The non-significant association between Home Food_DP_ and serum phosphorus level, despite higher protein intakes may be explained by the lower phosphate absorption from fruits and vegetables^17^. Achieving dietary protein adequacy without excessive phosphate intake is critical for MHD patients, yet challenging as phosphate is naturally found in protein-rich food. Shinaberger et al.^5^ showed that increased protein intake alongside with reduced serum phosphorus level was associated with better survival in MHD patients, but controlling serum phosphorus level by decreasing protein intake led to increased mortality. Thus the Home Food_DP_ reflecting a balanced diet with rice, protein foods, vegetables, and fruits, is clearly the answer to patients facing the phosphate-protein dilemma. In addition, we have previously shown that the Home Food pattern was associated with better nutritional status indicated by serum albumin, malnutrition inflammation score, and handgrip strength^14^.

The present study highlighted potential applications of the dietary pattern approach in nutritional intervention for MHD patients. The dietary pattern approach takes into account the multidimensional exposure and interactions that exist across complex combinations of dietary components and nutrients from foods^12^. As people consume foods instead of nutrients in isolation, dietary patterns can be readily translated into practical dietary advice. Nevertheless, we cannot refute the importance of recognizing and restricting inorganic phosphate intake. We have clearly shown that a dietary pattern containing beverages rich in inorganic phosphate was associated with hyperphosphatemia in this Malaysian population and this aspect should be communicated in patient education as well as incorporated into the local clinical practice guideline^8^. At the same time, it is also critical to emphasize to patients the value of a balanced diet.

There were some important limitations in this study. Firstly, this was a cross-sectional study with potential residual confounders and the causality cannot be established. Secondly, dietary intakes were assessed using the 3DDR method, which may be subject to measurement and recall bias. However, trained dieticians were involved in dietary data collection and expectedly minimized bias in recall data. Further, we excluded patients with implausible dietary data to minimize recall bias as per the standard protocol of dietary pattern analyses^15,32^, although this could also possibly lead to selection bias. The refusal rate for participation (37.9%) of this study could have also introduced selection bias. Thirdly, the process of dietary pattern derivation requires a high degree of subjective decision making such as aggregation of food items into food groups, the number of factors to retain, the method of rotation, and naming of patterns, which leads to inconsistency between researchers^46^. However, this limitation was overcome by consensus between researchers for the food listing and derivation of dietary patterns. A major limitation was the lack of organic and inorganic phosphate database or even its identification on processed food labels for us to accurately distinguish these two types of phosphate. Therefore, we had to use an assumption model to overcome this deficiency. Inclusion of parathyroid hormone as a parameter to examine phosphate resorption from bone and gut availability from dietary phosphate origin would have been ideal, but owing to its cost, we could not include this measurement for screening this population. The information on ultrafiltration volume was also not available in this study. Finally, this data-driven approach identified dietary patterns specific only to the study population and therefore, should not be generalized to other populations outside of Malaysia.

In conclusion, the SSB_DP_ was associated with higher concentrations of serum phosphorus in MHD patients, possibly due to greater intakes of inorganic phosphate. The Home Food_DP_ was associated with greater amount of protein intake, but was not associated with the serum phosphorus level. Therefore, evaluation of the habitual dietary pattern could provide valuable insights for the dietary management of hyperphosphatemia in MHD patients. Future studies investigating the effectiveness of dietary pattern intervention on hyperphosphatemia in MHD patients are warranted.

## Supplementary information

Supplementary Information 1.

## Data Availability

The datasets generated and/or analysed during this study are available on reasonable request from the corresponding author, T.K.
