# Supplementary material for: Association of dietary patterns with serum phosphorus in maintenance haemodialysis patients: a cross-sectional study
Source: Sci Rep. 2020 Jul 23;10:12278. doi: 10.1038/s41598-020-68893-4 (PMC7378243; doi:10.1038/s41598-020-68893-4)
Supplement: Supplementary file 1 — Supplementary Information 1. [file 41598_2020_68893_MOESM1_ESM.docx]

**Association of Dietary Patterns with Serum Phosphorus in Maintenance Haemodialysis Patients: A Cross-Sectional Study**

Ban-Hock Khor^1^, Ayesha Sualeheen^2^, Sharmela Sahathevan^2^, Karuthan Chinna^3^, Abdul Halim Abdul Gafor^1^, Sunita Bavanandan^4^, Bak-Leong Goh^5^, Zaki Morad^6^, Zulfitri Azuan Mat Daud^7^, Pramod Khosla^8^, Angela Yee-Moon Wang^9^, Tilakavati Karupaiah^10,*^, PaTCH Investigators

^1^Department of Medicine, Faculty of Medicine, Universiti Kebangsaan Malaysia, Cheras, 56000 Kuala Lumpur, Malaysia.

^2^Dietetics Program, Faculty of Health Sciences, Universiti Kebangsaan Malaysia, 50300, Kuala Lumpur, Malaysia.

^3^School of Medicine, Faculty of Health & Medical Science, Taylor’s University Lakeside Campus, Subang Jaya, 47500 Selangor, Malaysia.

^4^Department of Nephrology, Hospital Kuala Lumpur, 50586 Kuala Lumpur, Malaysia.

^5^Clinical Research Center, Hospital Serdang, 43000 Kajang, Selangor, Malaysia.

^6^National Kidney Foundation Malaysia, 46100 Petaling Jaya, Selangor, Malaysia.

^7^Department of Nutrition and Dietetics, Faculty of Medicine and Health Science, Universiti Putra Malaysia, Seri Kembangan, 43400 Selangor, Malaysia.

^8^Department of Nutrition and Food Science, Wayne State University, Detroit, MI, 48202 United States of America.

^9^Department of Medicine, Queen Mary Hospital, The University of Hong Kong, Hong Kong, China.

^10^School of BioSciences, Faculty of Health & Medical Science, Taylor’s University Lakeside Campus, Subang Jaya, 47500 Selangor, Malaysia.

*Correspondence: Tilakavati Karupaiah, School of Biosciences, Faculty of Health and Medical Sciences, Taylor’s University, Subang Jaya 47500, Malaysia. E-mail: [tilly_karu@yahoo.co.uk](mailto:tilly_karu@yahoo.co.uk)

**Table S1**. Comparison of variables between patients with normal serum phosphorus and hyperphosphatemia

|  | Normal serum phosphorus  (1.18 – 1.78 mmol/l)  (*n* = 180) | Hyperphosphatemia  (> 1.78 mmol/l)  (*n* = 208) | *P* value |
| --- | --- | --- | --- |
| Age (year) | 55.8 ± 13.1 | 53.3 ± 13.4 | 0.069 |
| Gender  Male  Female | 100 (46.3%)  80 (46.5%) | 116 (53.7%)  92 (53.5%) | 0.966 |
| Sector  Government  Non-governmental organization  Private | 77 (55.0%)  74 (39.8%)  29 (46.8%) | 63 (45.0%)  112 (60.2%)  33 (53.2%) | **0.024** |
| Ethnic  Malay  Chinese  Indian | 82 (50.0%)  66 (42.0%)  32 (47.8%) | 82 (50.0%)  91 (58.0%)  35 (52.2%) | 0.349 |
| Compliance to binder  Yes  No | 120 (53.3%)  59 (37.3%) | 105 (46.7%)  99 (62.7%) | **0.002** |
| Kt/V | 1.65 ± 0.35 | 1.61 ± 0.46 | 0.278 |
| Energy (kcal/kg) | 25.3 ± 6.9 | 25.0 ± 6.1 | 0.555 |
| Protein (g/kg)* | 52.2 (21.3) | 52.7 (21.2) | 0.820 |
| Phosphate (mg)* | 633 (286) | 631 (272) | 0.422 |
| Plant protein (g)* | 24.4 (10.0) | 23.4 (9.7) | 0.285 |
| Animal protein (g)* | 28.7 (19.1) | 28.9 (19.4) | 0.430 |
| Organic plant phosphate (mg)* | 277 (171) | 259 (146) | 0.099 |
| Organic animal phosphate (mg)* | 232 (199) | 250 (207) | 0.644 |
| Inorganic phosphate (mg)* | 86 (126) | 76 (153) | 0.688 |
| Phosphate protein ratio* | 12.1 (3.5) | 11.9 (3.2) | 0.131 |
| Home food_DP_  Tertile 1  Tertile 2  Tertile 3 | 50 (27.8%)  65 (36.1%)  65 (36.1%) | 75 (36.1%)  66 (31.7%)  67 (32.2%) | 0.219 |
| Sugar-sweetened beverages_DP_  Tertile 1  Tertile 2  Tertile 3 | 66 (36.7%)  58 (32.2%)  56 (31.1%) | 65 (31.3%)  64 (30.8%)  79 (38.0%) | 0.331 |
| Eating out-noodles_DP_  Tertile 1  Tertile 2  Tertile 3 | 100 (55.6%)  38 (21.1%)  42 (23.3%) | 116 (55.8%)  46 (22.1%)  46 (22.1%) | 0.947 |

Continuous data was presented as mean ± SD and independent t-test was used for analysis while categorical data was presented as frequency (%) and Chi-square test was used for analysis.

*Data was presented as median with interquartile range (IQR) and Mann-Whitney test was used for the analysis

**Table S2**. Associations between sociodemographic factors and dietary patterns

|  | Home Food_DP_ | | | |  | Eating-out Noodle_DP_ | | | |  | Sugar-sweetened Beverages_DP_ | | | |
| --- | --- | --- | --- | --- | --- | --- | --- | --- | --- | --- | --- | --- | --- | --- |
|  | Tertile 1 | Tertile 2 | Tertile 3 | *P* value |  | Tertile 1 | Tertile 2 | Tertile 3 | *P* value |  | Tertile 1 | Tertile 2 | Tertile 3 | *P* value |
| Age (year) | 54.5 ± 12.5 | 53.3 ± 13.6 | 56.0 ± 13.4 | 0.209 |  | 54.1 ± 13.6 | 54.1 ± 13.1 | 56.0 ± 13.1 | 0.427 |  | 58.5 ± 12.6 | 53.7 ± 13.7 | 51.8 ± 13.0 | <0.001^*^ |
|  |  |  |  |  |  |  |  |  |  |  |  |  |  |  |
| Gender |  |  |  |  |  |  |  |  |  |  |  |  |  |  |
| Male | 71 (29.7) | 79 (33.1) | 89 (37.2) | 0.249 |  | 119 (49.8) | 54 (22.6) | 66 (27.6) | 0.096 |  | 65 (27.2) | 73 (30.5) | 101 (42.3) | <0.001 |
| Female | 71 (36.2) | 65 (33.2) | 60 (30.6) |  |  | 83 (42.3) | 62 (31.6) | 51 (26.0) |  |  | 80 (40.8) | 72 (36.7) | 44 (22.4) |  |
|  |  |  |  |  |  |  |  |  |  |  |  |  |  |  |
| Ethnic |  |  |  |  |  |  |  |  |  |  |  |  |  |  |
| Malay | 65 (35.3) | 62 (33.7) | 57 (31.0) | 0.744 |  | 107 (58.2) | 52 (28.3) | 25 (13.6) | <0.001 |  | 44 (23.9) | 58 (31.5) | 82 (44.6) | <0.001 |
| Chinese | 52 (30.1) | 56 (32.4) | 65 (37.6) |  |  | 48 (27.7) | 46 (26.6) | 79 (45.7) |  |  | 79 (45.7) | 57 (32.9) | 37 (21.4) |  |
| Indian | 25 (32.1) | 26 (33.3) | 27 (34.6) |  |  | 47 (60.3) | 18 (23.1) | 13 (16.7) |  |  | 22 (28.2) | 30 (38.5) | 26 (33.3) |  |
|  |  |  |  |  |  |  |  |  |  |  |  |  |  |  |
| Sector |  |  |  |  |  |  |  |  |  |  |  |  |  |  |
| Govt | 58 (35.2) | 52 (31.5) | 55 (33.3) | 0.402 |  | 77 (46.7) | 45 (27.3) | 43 (26.1) | <0.001 |  | 45 (27.3) | 56 (33.9) | 64 (38.8) | 0.002 |
| Private | 27 (39.7) | 19 (27.9) | 22 (32.4) |  |  | 77 (38.1) | 59 (29.2) | 66 (32.7) |  |  | 86 (42.6) | 63 (31.2) | 53 (26.2) |  |
| NGO | 57 (28.2) | 73 (36.1) | 72 (35.6) |  |  | 48 (70.6) | 12 (17.6) | 8 (11.8) |  |  | 14 (20.6) | 26 (38.2) | 28 (41.2) |  |

Data is expressed as mean ± standard deviation or frequency (percentage)

Abbreviation: govt, government; NGO, non-governmental organization

**p*<0.05 for comparison between Tertile 1 vs Tertile 3
